# Chronic Inflammatory Demyelinating Polyneuropathy (CIDP) in Diabetes Mellitus: A Diagnostic Dilemma

**DOI:** 10.7759/cureus.25332

**Published:** 2022-05-25

**Authors:** Marcos Valentin, Ryan Coultas, Elisa Sottile

**Affiliations:** 1 Internal Medicine, University of Florida College of Medicine – Jacksonville, Jacksonville, USA

**Keywords:** peripheral motor neuropathy, electromyography and electro-stimulation, nerve conduction study (ncs), type-2 diabetes mellitus, chronic inflammatory demyelinating neuropathy

## Abstract

Chronic inflammatory demyelinating polyneuropathy (CIDP) is a neurological disorder of the peripheral nerves which can lead to gradually increasing motor and sensory loss. It can be a difficult entity to diagnose, particularly in elderly patients with a history of Diabetes Mellitus due to their overlapping neuropathic syndromes. Reported is a case of CIDP in an elderly female who manifested multiple sensory, motor, and autonomic complaints. A compilation of clinical features, neuroimaging, lumbar puncture, electromyography, nerve conduction studies, and nerve biopsy were used to reach the diagnosis. Highlighted is a clinical approach to identifying CIDP that can cause neuropathy in the setting of other potential confounding disorders namely Diabetes Mellitus.

## Introduction

Chronic inflammatory demyelinating polyneuropathy (CIDP) is a rare form of acquired peripheral neuropathy that can present with a wide variety of sensory and motor deficits. In the most typical form, there is gradually progressive, symmetric loss of distal and proximal motor function as well as sensory involvement that is less prominent in comparison [1]. We report a complex case of CIDP involving a 74-year-old female who developed severe autonomic, sensory, and motor complaints leading up to her diagnosis.

## Case presentation

A 74-year-old female with Insulin-dependent Type II Diabetes Mellitus (DM) and a history of gastric bypass surgery presented due to progressive motor decline over a four-month period. Her history was notable for a neurogenic bladder diagnosed two years prior and chronic pain of the neck, shoulders, hips, and lower back. Her weakness progressed in an ascending pattern, initially involving the lower extremities with associated foot drop, and recurrent falls followed by involvement of the arms and bulbar weakness causing severe oropharyngeal dysphagia. On presentation, she was found to have marked atrophy and weakness involving the thigh and calve musculature. Deep tendon reflexes were globally diminished. Babinski's reflex was negative. Sensory testing revealed allodynia, decreased vibratory sense, normal light touch sense, and decreased proprioception in the lower extremities.

**Figure 1:**
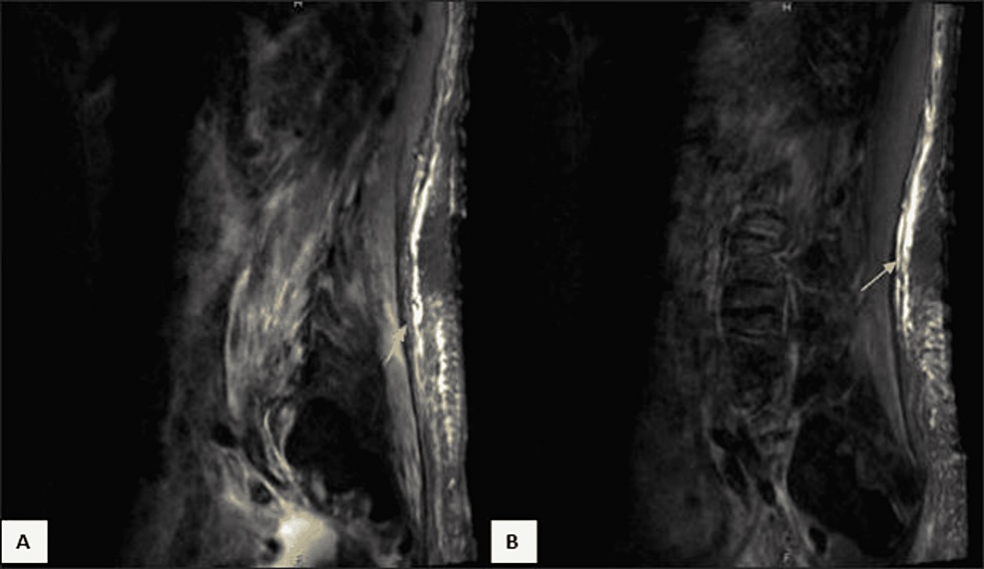
Sagittal STIR weighted image on MRI Sagittal short TI inversion recovery (STIR) weighted image on MRI showing hyperintense signal (arrow) of the right (A) and left (B) paraspinal musculature.

**Figure 2:**
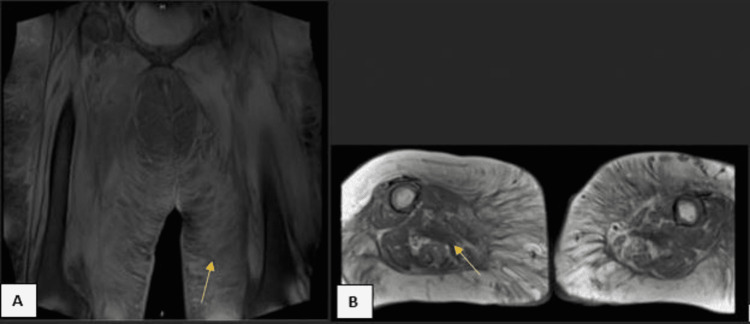
Proton density, fat saturation images of the bilateral thighs Proton density, fat saturation coronal (A) and T1 weighted axial (B) views of the bilateral thighs showing diffuse subcutaneous edema and sarcopenia.

Serologic testing including vitamin and trace mineral levels, creatinine kinase, aldolase, paraproteins, antinuclear antibodies (ANA), thyroid function, and acetylcholine receptor antibodies were all unremarkable. Her hemoglobin A1c was 5.9%. Magnetic resonance imaging (MRI) of the cervical, thoracic, and lumbosacral spine showed multilevel disc disease with mild-moderate neuroforaminal narrowing affecting the C5, C6, and L2 nerve roots and hyperintense short TI inversion recovery (STIR) signals within the paraspinal musculature (Figure 1A, 1B). MRI of the bilateral thighs showed diffuse sarcopenia and subcutaneous edema (Figure 2A, 2B).

A lumbar puncture (LP) resulted in an opening pressure of 15 cmH_2_O (6-2 cmH_2_O), protein of 187 mg/dL (15-45 mg/dL), glucose of 39 mg/dL (60-80 mg/dL), and white blood cell count of 3 x 10^6^/L. Electromyography (EMG) and Nerve conduction studies (NCS) are depicted in Tables 1-4. Results showed absent bilateral median, ulnar, and right radial sensory responses. Absent bilateral peroneal and tibial motor responses. Left median and ulnar motor distal latencies were within normal limits with reduced compound motor action potential (CMAP) amplitudes (proximal stimulation not obtained due to dressing present in the arm). Right median and ulnar motor distal latencies were within normal limits with reduced CMAP amplitudes and reduced motor conduction velocity. Left median and ulnar F-wave latency near normal upper limit. Right ulnar F-wave latency was slightly prolonged. Right median F-wave latency was normal. Overall, this study did not show evidence for myopathy or demyelinating features but was limited due to not being able to obtain motor nerve conduction responses in the lower extremities.

**Table 1:** Sensory NCS NR - No Response; NCS - Nerve conduction studies

Nerve/Sites	Recorded Site	Latency ms	Amplitude mV
Left Median - Digit II (Ortho)			
Digit II	Wrist	NR	NR
Left Ulnar - Digit V (Ortho)			
Digit V	Wrist	NR	NR
Right Ulnar - Digit V (Ortho)			
Digit V	Wrist	NR	NR
Right Radial - Snuff			
Forearm	Wrist	NR	NR

**Table 2:** Motor NCS APB - Abductor pollicis brevis; ADM - Abductor digiti minimi; EDB - Extensor digitorum brevis; AHB - Abductor hallucis brevis; NR - No response; NCS - Nerve conduction studies

Nerve/Sites	Recorded Site	Latency ms	Amplitude mV	Distance cm	Velocity m/s
Left Median - APB					
Wrist	APB	4.43	1.8		
Right Median - APB					
Wrist	APB	3.91	2.4		
Elbow	APB	9.9	2.0	25	42
Left Ulnar - ADM					
Wrist	ADM	2.97	2		
Right Ulnar - ADM					
Wrist	ADM	3.49	0.9		
Below Elbow	ADM	8.65	0.9	24	47
Above Elbow	ADM	10.47	0.9	10	55
L Peroneal - EDB					
Ankle	EDB	NR	NR		
L Tibial - AHB					
Ankle	AHB	NR	NR		
R Peroneal -EDB					
Ankle	EDB	NR	NR		
R Tibial - AHB					
Ankle	AHB	NR	NR		

**Table 3:** F Wave APB - Abductor policis brevis; ADM - Abductor digiti minimi

Nerve	F Min ms
Left Median - APB	32.7
Left Ulnar- ADM	32.8
Right Median - APB	31.7
Right Ulnar - ADM	34.7

**Table 4:** Needle EMG EMG - Electromyography

	Spontaneous				Motor Unit Potential			Recruitment
Muscle	IA	Fib/PSW	Fasc	Misc	Amplitude	Duration	Phases	Pattern
Right Tibialis anterior	Normal	0	0	0	Normal	Normal	Increased	Sub Max
Right Vastus medialis	Normal	0	0	0	Normal	Normal	Increased	Sub Max
Right Vastus lateralis	Increased	1+	0	0	Normal	Normal	Normal	Sub Max
Left Vastus lateralis	Normal	0	0	0	Normal	Normal	Increased	Sub Max
Right Deltoid	Normal	0	0	0	Normal	Normal	Normal	Sub Max
Right Triceps brachii	Normal	0	0	0	Normal	Normal	Normal	Normal
Right First dorsal interosseous	Normal	0	0	0	Normal	Normal	Increased	Sub Max

The patient underwent quadriceps muscle and sural nerve biopsy which showed marked muscle atrophy and extensive loss of large and small myelinated nerve fibers with features of active axonal and demyelinating neuropathy. There were rare endoneurial T-Lymphocytes and a macrophage attached to relatively intact myelin of one nerve fiber. There was no onion bulb formation. Also noted were arteriosclerotic changes without evidence of vasculitis. A history of progressive weakness coupled with findings of cytoalbuminologic dissociation on LP and evidence for demyelination on biopsy was most consistent with a diagnosis of CIDP. The patient was initiated on intravenous (IV) solumedrol 250 mg daily for three days then transitioned to 1 mg/kg daily. She was then treated with intravenous immunoglobulin (IVIG) 2 g/kg evenly distributed over a period of five days. She experienced an overall improvement in her upper extremity and bulbar weakness. Steroids were tapered over a two-month period. Her lower extremity weakness had mild improvement, but she remained wheelchair dependent. She was arranged for outpatient IVIG 1 g/kg daily over two days every three weeks as maintenance.

## Discussion

CIDP is a type of immune-mediated neuropathy that can lead to progressive weakness, abnormal sensation, and autonomic dysfunction. The diagnosis of CIDP can be challenging to ascertain given its rarity and similarities with other common neuropathic diseases. History of DM is especially problematic when considering CIDP given that diabetic polyneuropathy and diabetic amyotrophy can also lead to elevated protein in the cerebrospinal fluid (CSF) and axonal damage as seen in nerve conduction studies and nerve biopsy. Nutritional deficiency and insulin neuritis were also considered but difficult to prove in retrospect. A normal hemoglobin A1c and response to treatment favors CIDP over diabetic neuropathy in our case but does not effectively rule it out. Interpretation of electromyography (EMG) and nerve conduction studies in the presence of multilevel intervertebral disc disease also poses a challenge. Despite the multifactorial nature of her disease, a diagnosis of CIDP can still be made based on clinical characteristics, electrophysiological criteria, biopsy, and CSF findings.

No gold-standard set of diagnostic criteria exists, however, the European Federation of Neurological Societies and the Peripheral Nerve Society (EFNS/PNS) criteria seem to be one of the most useful in identifying CIDP with a reported sensitivity and specificity of 81 and 97% respectively [2]. The EFNS/PNS guideline defines criteria for typical and atypical CIDP that are based on clinical, electrodiagnostic, and supportive criteria.

Typical CIDP is defined by chronically progressive, stepwise, or recurrent symmetric proximal and distal weakness and sensory dysfunction of all extremities, developing over at least two months. There are also absent or reduced tendon reflexes in all extremities. Other causes of demyelinating neuropathy must be ruled out such as POEMS syndrome, Lyme infection, or lumbosacral radiculoplexus neuropathy. Other supportive criteria are defined including cytoalbuminologic dissociation on CSF, characteristic neuroimaging findings, abnormal sensory electrophysiology, objective improvement following immunomodulatory treatment, and nerve biopsy showing evidence of demyelination [3]. Other forms of CIDP are characterized by the predominance of sensory involvement or the identification of autoantibodies against nodal or paranodal proteins. Autonomic dysfunction is an uncommon feature of CIDP and is more prominent as a complication of diabetes when present. 

MRI with gadolinium is the imaging modality of choice and should be performed to exclude other forms of neuropathy that can mimic CIDP. Features that are suggestive of CIDP are thickening and enhancement of peripheral nerves, brachial or lumbosacral plexus, and nerve roots [4].

Electrodiagnostic testing is an essential component of diagnosing CIDP as most patients will show evidence of primary demyelination. Features that are suggestive of demyelination on nerve conduction and electromyography testing include partial conduction block, conduction velocity slowing, temporal dispersion, and distance-dependent reduction of CMAP [5]. It is however worth noting that other nerve diseases like diabetic neuropathy can also evoke similar findings. 

Some studies even suggest a potential association between CIDP and DM. In some cohort analyses, diabetics were observed to meet electrophysiologic criteria for CIDP 12-17% of the time [6,7]. The prevalence of CIDP also tends to be higher in diabetics compared to nondiabetics [8]. CIDP in diabetics however tends to present in older patients and more in the typical form compared to idiopathic CIDP [9]. The axonal loss also tends to be more severe in patients with concurrent CIDP and DM which likely confers worse outcomes following treatment [10]. Biopsy does not distinguish diabetic neuropathy from CIDP as both can show varying amounts of demyelination and axonal loss. Histological analysis showing an inflammatory infiltrate, prominent demyelination, or onion bulb formation can be highly suggestive of CIDP but only seen in a minority of cases. Still, it is worth noting that CIDP is a treatable disease. In some cases, assessing response to immunotherapy can aid in confirming a diagnosis of CIDP retrospectively whereas diabetic polyneuropathy should not respond to such treatment [11]. 

There are no laboratory findings that are specific to CIDP, however, certain tests should be done to exclude other disorders. In general laboratory testing should include complete blood count, liver function tests, thyroid function studies, serum and urine protein electrophoresis with immunofixation, serum-free light chain assay, fasting serum glucose, glycated hemoglobin, and serum calcium and creatinine. CSF analysis revealing an elevated protein level is a nonspecific finding but tends to be higher in CIDP compared to diabetic neuropathy.

## Conclusions

This case highlights the complexity of diagnosing CIDP, particularly in the presence of multiple comorbid conditions which can confound clinical, imaging, and electrophysiologic studies. It is important to remember that CIDP is a treatable form of neuropathy in diabetics. Clinicians should have a low index of suspicion for CIDP in diabetics with progressive motor neuropathy that is unproportionate to that of diabetic neuropathy alone and to weigh the potential benefit of treating these patients with immunotherapy. Though a primarily motor neuropathy, it is also important to recognize the wide heterogeneity of CIDP types and to not discount this diagnosis based on atypical features of dysautonomia and sensory dysfunction.
